# Leukocyte counts in urine reflect the risk of concomitant sepsis in bacteriuric infants: A retrospective cohort study

**DOI:** 10.1186/1471-2431-7-24

**Published:** 2007-06-13

**Authors:** Bema K Bonsu, Marvin B Harper

**Affiliations:** 1 Department of Pediatrics, Division of Emergency Medicine, Columbus Children's Hospital, OH, USA; 2 Department of Medicine, Divisions of Emergency Medicine and Infectious Diseases, Children's Hospital, Boston, MA, USA

## Abstract

**Background:**

When urine infections are missed in febrile young infants with normal urinalysis, clinicians may worry about the risk – hitherto unverified – of concomitant invasion of blood and cerebrospinal fluid by uropathogens. In this study, we determine the extent of this risk.

**Methods:**

In a retrospective cohort study of febrile 0–89 day old infants evaluated for sepsis in an urban academic pediatric emergency department (1993–1999), we estimated rates of bacteriuric sepsis (urinary tract infections complicated by sepsis) after stratifying infants by urine leukocyte counts higher, or lower than 10 cells/hpf. We compared the global accuracy of leukocytes in urine, leukocytes in peripheral blood, body temperature, and age for predicting bacteruric sepsis. The global accuracy of each test was estimated by calculating the area under its receiver operating characteristic curve (AUC). Chi-square and Fisher exact tests compared count data. Medians for data not normally distributed were compared by the Kruskal-Wallis test.

**Results:**

Two thousand two hundred forty-nine young infants had a normal screening dipstick. None of these developed bacteremia or meningitis despite positive urine culture in 41 (1.8%). Of 1516 additional urine specimens sent for formal urinalysis, 1279 had 0–9 leukocytes/hpf. Urine pathogens were isolated less commonly (6% vs. 76%) and at lower concentrations in infants with few, compared to many urine leukocytes. Urine leukocytes (AUC: 0.94) were the most accurate predictors of bacteruric sepsis. Infants with urinary leukocytes < 10 cells/hpf were significantly less likely (0%; CI:0–0.3%) than those with higher leukocyte counts (5%; CI:2.6–8.7%) to have urinary tract infections complicated by bacteremia (N = 11) or bacterial meningitis (N = 1) – relative risk, 0 (CI:0–0.06) [RR, 0 (CI: 0–0.02), when including infants with negative dipstick]. Bands in peripheral blood had modest value for detecting bacteriuric sepsis (AUC: 0.78). Cases of sepsis without concomitant bacteriuria were comparatively rare (0.8%) and equally common in febrile young infants with low and high concentrations of urine leukocytes.

**Conclusion:**

In young infants evaluated for fever, leukocytes in urine reflect the likelihood of bacteriuric sepsis. Infants with urinary tract infections missed because of few leukocytes in urine are at relatively low risk of invasive bacterial sepsis by pathogens isolated from urine.

## Background

Urinary tract infection is by far the most common serious bacterial infection in febrile young infants [1-8]. The diagnosis is suspected when screening urinalysis reveals pyuria and confirmed when a uropathogen is isolated from urine culture. When, however, this infection is missed on account of few leukocytes in urine (a recognized limitation), there is the concern – empirically unsubstantiated at present – that affected infants left untreated at the initial clinical encounter will return with invasive sepsis.

For this reason, the challenge to clinicians when utilizing screening urinalysis – heightened when relying on this test alone for decision-making (an option endorsed by clinical practice guidelines for the management of fever in young well-appearing infants [9]) – is to determine not only that the test is able to identify urinary tract infections, but also that it independently reflects the risk, at the initial visit, of invasive bacterial infections due to the urinary tract pathogen if present. To date, few data have been published regarding the utility of the screening urinalysis in this role. In this study, we tackle this question, hypothesizing an association in febrile young infants between the concentration of leukocytes in urine and the risk of bacteriuric sepsis.

## Methods

We conducted a retrospective cohort study of consecutive febrile (temperature in triage ≥ 38°C) infants aged 0 days to 89 days seen in the emergency department at Children's Hospital Boston from January 1993 to June 1999. This 6-year period immediately followed, (1) the publication in 1993 of practice guidelines for fever in young children and infants [9] and (2) the formal adoption, specifically at our institution, of a novel strategy for routine and comprehensive management of febrile young infants on an outpatient basis [6]. It reflects, therefore, an interval during which compliance with full laboratory testing to detect bacterial sepsis – an integral part of this strategy – was likely to be highest (unlikely to have become degraded by the passage of time), mitigating the potential for test selection bias [9]. Ethical approval to conduct the study was granted by the Institutional Review Board of the hospital and consent by patients was not required.

The emergency department is located within an academic children's hospital in an urban setting. Families are either self-referred (elect to come to the emergency department) or are referred by their primary care physicians. Annually, there are 50,000 visits to the department. Thirty-five percent of patients visiting the department are Caucasian, 25% are African American, 20% are Hispanic, and 20% constitute other races or else did not state their racial origin. Patients included in the study were young infants presenting to our emergency department with fever who underwent a full sepsis workup. This sepsis strategy comprises collection of urine, blood, and cerebrospinal fluid for screening and culture. Screening test results of urine (dipstick/urinalysis), blood (white blood cell count), and spinal fluid (white blood cell count) are promptly reported to the treating physician on the day of encounter and used to inform decisions as to whether to admit or discharge, as well as whether to administer or withhold antibiotic treatment.

Screening dipstick was performed using Multistix (Bayer Corporation, Elkhard, IN). Per protocol at the institution, the laboratory performed microscopic analysis of urine (formal urinalysis) only if the dipstick examination was positive for nitrite, blood, leukocyte esterase, or protein. All urine samples, however, were sent for bacterial culture irrespective of urine dipstick findings. The leukocyte count reported on microscopic analysis was measured as the number of cells per high power field (hpf, 40× magnification) in urine centrifuged at 2000 revolutions per minute for 5 minutes. Results were reported on an extended ordinal scale that we abbreviated to three categories: 0–9, 10–50 and greater than 50 leukocytes per hpf in urine. Microscopy by standard urinalysis technique was applied equally to all urine specimens sent for formal urinalysis during the period of study.

Results of urine bacterial culture were recorded for each febrile young infant seen at this site. We defined positive urine culture based on published thresholds (colony forming units per ml [cfu/ml]) for each urine collection technique [4,10]. Urine obtained by bladder catheterization was considered positive if a known pathogen was isolated at concentrations greater than or equal to 10,000 cfu/ml. Specimens obtained by supra-pubic aspiration were considered positive if a known pathogen was isolated at concentrations greater than or equal to 1000 cfu/ml. Specimens collected by clean void or by unknown technique were considered positive only if the concentration of bacteria in urine was greater than or equal to 100,000 cfu/ml. Urine that isolated a known contaminant was considered not to be infected but was retained in the dataset.

For young infants growing a pathogen from urine, culture results of blood and cerebrospinal fluid specimens obtained concurrently during the initial encounter were examined for evidence of concomitant infection by the same pathogen. Concomitant (also designated invasive or bacteriuria-associated) bacteremia and bacterial meningitis were diagnosed if standard culture of blood or cerebrospinal fluid grew the same pathogen found in urine. Cases in which bacteremia or bacterial meningitis were diagnosed in infants with no evidence of urinary tract infection were designated non-bacteriuric sepsis.

### Analysis

For our primary analysis, we determined the relative risk (with 95% confidence limits) of concomitant sepsis between bacteriuric infants with low as opposed to high concentrations of leukocytes in urine. Specifically, we compared the frequency of urinary tract infections associated with concomitant bacteremia or bacterial meningitis between infants that had 0–9 leukocytes/hpf versus > 9 leukocytes/hpf on confirmatory urine microscopy. For suitable parts of our analysis, we also combined infants with 0–9 leukocytes in urine with those that had negative dipstick results. We hypothesized that the risk in febrile young infants of urinary tract infections complicated by invasive sepsis would differ significantly depending on whether leukocytes in urine were few or many.

The accuracy of urine leukocytes for predicting concomitant bacteremia and bacterial meningitis was determined by estimating the area under receiver operator characteristic curves (AUC) for this test. The AUC is a summative index of true to false positives for a given outcome at ordered values of a diagnostic test. An AUC value of 0.5 indicates no value of a test for diagnosis. A value of 1.0, on the other hand, shows the test is able to identify the target outcome perfectly. We adopted an AUC cut-point of 0.7 as the minimum threshold for designating a test to be useful for diagnosis [11,12].

In a secondary analysis, we determined the diagnostic accuracy of other variables that have the potential to be predictors of invasive sepsis, concentrating on those that are measured easily. Specifically, we estimated AUC values for body temperature and age (both recorded routinely at triage), as well as for peripheral blood leukocytes (easily obtained from blood specimens collected by finger stick). Based on previous studies, we estimated the AUC value for non-linear (J-shaped) diagnosis models of peripheral blood leukocytes [13,14].

Finally, among infants with positive urine culture, we gauged whether groups stratified by the leukocyte count in urine differed sufficiently in respect to other markers of infection to permit assignment to separate clinical or prognostic groups. Specifically, we compared the concentration of bacteria in urine culture, the frequency of hematuria (> 5 red blood cells/hpf) and the fraction with positive nitrites in urine at the initial visit among infants with leukocytes in urine greater versus fewer than 10 cells/hpf.

Data were analyzed with the Stata statistical package (version 8.0, Stata Corp., TX). The chi-square and Fisher exact tests were used to compare count data. Medians of data that were not normally distributed were compared by the Kruskal-Wallis test.

## Results

For the period of study, urine samples were obtained from 3765 febrile infants aged 0–89 days presenting to Children's Hospital Boston and sent for screening urine dipstick and bacterial culture. Of these, positive urine culture was reported in 307 (8.2%). Urine was sent for formal microscopy after dipstick analysis in 1516 (40%) of 3756 infants. Of these, 258 (17%) met study criteria for positive culture.

Seventy-nine of 258 pathogens (75 by bladder catheterization, 1 by clean void, and 3 by unknown technique) were isolated from urine specimens with 0–9 leukocytes/hpf at the initial visit. These included: *E. coli *(61), *Enterococcus *spp (7), *Enterobacter *spp (5), *Klebsiella pneumoniae *(4) and *Citrobacter *spp (2).

Organisms isolated from 179 cases with greater than or equal to 10 leukocytes/hpf in urine included *E. coli *(169), *Klebsiella pneumoniae *(3), *Pseudomonas aeruginosa *(2), Enterococcus spp (2), *Staphylococcus aureus *(1), *Citrobacter *spp (1), and *Enterobacter *spp (1). One hundred seventy of these pathogens were cultured from urine obtained by bladder catheterization, 4 by clean void, and 5 by unknown technique.

### Urine leukocytes as predictors of urinary tract infections

The rate of positive urine culture increased with rising concentrations of urinary leukocytes in these infants: 6.2% (79/1279) for infants with 0–9 leukocytes/hpf, 68% (88/130) for infants with 10–50 leukocytes/hpf, and 85% (91/107) for those with more than 50 leukocytes/hpf. Twelve pathogens isolated from urine were also cultured from blood or cerebrospinal fluid. These included 10 cases of *E. coli *bacteremia, 1 case of *S. aureus *bacteremia, and 1 case of meningitis caused by *E. coli*.

### Urine leukocytes as predictors of bacteriuric sepsis

The risk of concomitant sepsis (urinary tract infections complicated by bacteremia or meningitis) varied significantly between infants with 0–9 and those with greater than 9 leukocytes/hpf in urine at the initial visit. Among 1279 infants with 0–9 leukocytes/hpf, none (0%, 97.5% confidence intervals: 0–0.3) had concomitant bacterial infections. In contrast, all 12 cases of invasive sepsis (5.1%; 95%CI: 2.6–8.7) occurred in 237 infants with urine leukocyte counts above 9 cells/hpf. These differences translated to a relative risk of invasive sepsis of 0 (95%CI: 0–0.06) among cases with low versus high concentrations of leukocytes in urine. All 12 cases of invasive bacteremia and meningitis occurred in infants that had bacterial concentrations in urine that exceeded 100,000 cfu/ml.

In 2249 young infants, urine microscopy was not performed because they had negative urine dipstick results. Only 41 (1.8%) of these infants grew a pathogen from culture of urine. None (0%, 97.5% upper CI: 0.2%) had concomitant bacteremia or meningitis. When these infants were combined with infants who had 0–9 leukocytes/hpf, none of 3528 (0%, 97.5% upper CI: 0.1%) had concomitant sepsis – including 0 of 41 bacteriuric infants with negative dipstick (no urine microscopy) and 0 of 258 bacteriuric infants with 0–9 leukocytes/hpf, for a rate of 0 of 299 (0%, 97.5% upper CI: 1.2%) – translating to a relative risk of sepsis (compared to infants with pyuria) of 0 (95%CI: 0–0.02). The rate of non-bacteriuric sepsis (sepsis without associated bacteriuria) was equally low (0.8%) in both groups.

### Other potential predictors of bacteriuric sepsis

For identifying young infants at risk of concomitant bacteriuric sepsis at the initial encounter, the leukocyte count in urine when compared to other potential predictors was more accurate than age, temperature, peripheral bands and leukocytes in blood (specifications of the non-linear model for leukocytes in peripheral blood are provided in the appendix). Specifically, the AUC value for urine leukocytes, at 0.94, was higher than that estimated for temperature (0.52), age (0.62), leukocytes in blood (0.64), and peripheral bands (0.78).

### Other differences noted between bacteriuric infants with low versus high leukocyte counts in urine

Finally, among infants subsequently reported to have a positive urine culture, there were additional differences observed – besides the risk of invasive sepsis – between those with low and high urine leukocytes, supporting categorization to separate groups, Table 1. Specifically, among 245 (95%) febrile infants with specific notation in the medical chart of urine collected by bladder catheterization or supra-pubic tap, concentrations of bacteria in urine, as well as rates of hematuria, and proportions with positive urine nitrites differed significantly between the 2 groups. Concentrations of bacteria in urine differed significantly, both when compared directly (non-parametrically) and after collapsing the data from a semi-quantitative scale to a binary scale (being, the number of specimens with bacteria > 100,000 cfu/ml, Table 1).

**Table 1:** A comparison of demographic, clinical, and laboratory findings between bacteriuric infants with low vs. high leukocytes in urine

Variable	75 infants with 0–9 leukocytes/hpf in urine: Median: IQR*; or Percentages, 95%CI (N)	170 infants with ≥ 10 leukocytes/hpf in urine: Median: IQR*; or Percentages, 95%CI (N)
Gender (% Male)	60: 48–71 (45/75)	56: 49–64 (96/170)
Race (% Caucasian)	37:26–49 (28/75)	42: 34–50 (71/170)
Age (months) *	1.8: 1.0–2.3	1.4: 0.9–2.2
Triage temperature/°C *	38.6: 38.3–39.3	38.9: 38.5–39.5
Grades 4–5 VUR (%) §	3.2: 0.4–11.1 (2/62)	5.9: 2.7–10.9 (9/153)
Peripheral blood leukocyte count (cells/mm^3^) *	14,700: 10,720–19,230	15,300: 11,000–19,900
Cerebrospinal fluid leukocyte count (cells/mm^3^) *	4: 2–7	4: 2–8
Bladder catheterization (%)	83: 72–90 (62/75)	90: 84–94 (153/170)
Positive urine bacterial nitrite (%)	20: 12–31 (15/75)†	46: 38–54 (78/170)†
Hematuria: > 5 red blood cells in urine/hpf (%)	7: 2–17 (4/57)†	33: 26–41 (51/155)†
Bacterial counts in urine culture ≥ 100,000 cfu/ml (%) ‡	72: 60–82 (54/75)†	93: 88–96 (158/170)†
Bacteremia and/or bacterial meningitis (%)	0: 0–4 (0/75)†	7: 4–12 (12/170)†

* Inter-quartile range for median values (otherwise, 95% confidence intervals for percentages with numbers). †Significant at a nominal p value < 0.05. ‡ Colony forming units per ml of a single bacterium in urine culture. §Vesico-ureteral reflux.

## Discussion

Screening urinalysis is only moderately sensitive and has been reported to miss approximately 20% of urinary tract infections in febrile young infants [3,5-8,10,15-22]. The worry, when this occurs, is that infants with missed urinary tract infections on account of normal screening urinalysis results will progress to septicemia or bacterial meningitis from concomitant bacteremia unrecognized at the initial visit. We show in this study that this concern is unwarranted for the most part because urine leukocytes are an adequate screen for bacteriuric sepsis. Specifically, we find that few leukocytes in urine and a negative urine dipstick result at the initial visit reflect a low risk of concomitant sepsis in young infants when a urinary pathogen is unexpectedly isolated from culture.

Other plausible markers of bona fide urinary tract infections such as the concentration of bacteria in urine also differ significantly between bacteriuric infants with low and high leukocytes in urine, supporting categorization to separate clinical groups. Based on these differences and the relatively low risk of concomitant sepsis observed in the present study, it appears safe to manage, on an outpatient basis, febrile young infants who are well-appearing and have few leukocytes in urine, as recommended in clinical practice guidelines for fever [9]. The risk of adverse outcome when depending on this strategy is likely to be low provided infants are carefully selected for outpatient management, the interval to urine culture result is short, follow-up is assured, and other tests, when deemed necessary, are available for the complementary but separate task of identifying infants with serious bacterial infections not associated with bacteriuria [15].

Our findings corroborate those by other investigators who have also reported differences in clinical outcomes between infants and children with urinary tract infections after stratification by the leukocyte count in urine [2,22-24]. Of particular interest, are studies that report lower levels of acute phase reactants in blood, fewer structural abnormalities of the urinary tract, and a lower incidence of renal scarring, suggesting milder disease in children less than 24 months of age with few leukocytes in urine [22,23]. The specific reasons are unclear for the outcomes noted in these studies and ours between young infants with low and high urine leukocyte counts. It is conceivable, however, that few urine leukocytes in infants diagnosed with urinary tract infections reflect low virulence of pathogens, indicate mere colonization or contamination of urine, suggest infection confined to the lower urinary tract, or point to urine infections of insufficient duration or severity to induce an inflammatory response. The lower concentration, observed in our study, of bacteria isolated from urine of infants with normal urinalysis supports a number of these hypotheses. Unfortunately, there is currently no timely way to differentiate between these possibilities in a way that is accurate enough to have clinical utility at the initial visit.

To inform initial management decisions, therefore, it seems to us more pragmatic to identify in operational terms, as done in this study, febrile well-appearing infants at risk of bacteriuric sepsis. Risk-stratification for sepsis that is based on the urine leukocyte count undergirds expectant fever management strategies and is supported by studies that indicate not only a low risk to withholding empiric antibiotic treatment, but also the non-trivial prospect of developing acute pyelonephritis within months of antibiotic treatment in young children with few leukocytes in urine ultimately diagnosed with asymptomatic bacteriuria [25-28]. The low odds, observed in this study, of invasive sepsis complicating unrecognized bacteriuria in febrile infants with few leukocytes in urine, complements in the acute setting observations previously noted at longer time intervals. Together, these data reinforce fever management strategies that rely on screening tests like urinalysis for decision-making vis-à-vis supporting more judicious antibiotic treatment in febrile well-appearing young infants (specifically, those beyond the neonatal period) who satisfy low-risk criteria for sepsis.

Our study has a number of limitations. First, being retrospective, it shares the limitations of this study design. Primarily, we cannot prove a pathophysiological link between the urine leukocyte count and the likelihood of invasive bacterial infections. It is also not possible to determine, retrospectively, whether antibiotics were administered prior to urine collection in some infants. Fortunately, at our site, antibiotic pretreatment in this age-group is highly unlikely and so would not alter our conclusions. The enhanced urinalysis technique (compared to standard urinalysis), because of better test performance characteristics, may support more accurate diagnosis but was not evaluated by us because it is not utilized at our institution [3]. Irrespective of the specific technique utilized, however, it is reasonable to expect an even lower urine leukocyte count to translate to still lower odds of concomitant bacteriuric sepsis in infants with initially missed urinary tract infections. With respect to standard urinalysis, some clinicians may find it useful to lower the threshold for urine leukocytes to 5 cells/hpf – as proposed in a recent study [4] – to further reduce the risk of bacteriuric sepsis associated with expectant management. As mentioned earlier, test selection bias was unlikely to affect our results appreciably during the years of study, an interval during which compliance with full sepsis protocols was likely to be highest.

Finally, although not specifically evaluated in this study, we expect the short delay reported in a recent study from the first visit to a report of positive urine culture – about 16 hours [29] – to further lower the risk of invasive sepsis and to permit the safe application of expectant strategies in febrile young infants. Rapid turn-around of the urine culture report should shorten the exposure of infants to unduly long periods of unrecognized urinary tract infection, concomitant sepsis, or needless antibiotic treatment. In cases among whom fever is merely the first sign of an incipient but easily recognized viral infection [30-35], expectant management may also permit a firmer diagnosis of asymptomatic bacteriuria to be made. Prospective studies are needed to formally investigate these possibilities and to solidify our results. We do not address the related issue of the impact of urinary leukocytes on the risk of renal scarring among young infants with positive urine culture. Other investigators address this outcome more fully, reporting a low risk in infants and young children with low leukocytes in urine [22,23].

## Conclusion

In conclusion, we show that urine leukocytes reflect well the risk of concomitant sepsis in febrile young (0–89 day old) infants with bacteriuria. Reassuringly, we find low odds of invasive sepsis by uropathogens even when urinary tract infections – the most common serious bacterial infections at this age – are missed at the initial visit on account of few leukocytes in urine. The low risk of invasive sepsis in bacteriuric infants with 'normal' screening urinalysis mitigates a major limitation of this test – a modest tendency to miss urinary tract infections – and indicates that fever management strategies reported in the literature that rely on urinalysis for screening are likely to be safe in selected febrile well-appearing young infants managed on an outpatient basis and assured of close follow-up.

## Competing interests

The author(s) declare that they have no competing interests.

## Authors' contributions

BKB and MBH both made substantial contributions to the conception, design, execution and completion of the study. BKB conceived the idea and designed the study in collaboration with MBH. MBH acquired the data and obtained permission from the ethical board of the institution to conduct the study. BKB performed the initial statistical analysis. Both BKB and MBH reviewed the analysis and interpretation of data. BKB drafted the manuscript, and together with MBH revised it critically for important intellectual content. Both BKB and MBH have read and approve the final manuscript.

## Appendix

Specifications of non-linear (J-shaped) models of leukocytes (WBC) in peripheral blood for predicting the log odds of invasive sepsis:

Log odds of invasive sepsis = -4.3 - 5.2 [ln (WBC ÷ 10^4^) - 0.2] + 10 [(WBC ÷ 10^4^)^0.5 ^- 1.1)]

These models represent the natural log odds of invasive sepsis in febrile young infants as a function of the leukocyte count in peripheral blood. To calculate the natural log odds of this outcome in a particular patient, insert the specific peripheral blood leukocyte count into the equation. Conversion to odds is then obtained by calculating the exponent of the numerical value obtained for the equation of the natural log odds of invasive sepsis.

## Pre-publication history

The pre-publication history for this paper can be accessed here:


